# Supplementary material for: Association between COVID-19 Vaccines and Menstrual Disorders: Retrospective Cohort Study of Women Aged 12–55 Years Old in Catalonia, Spain
Source: Int J Environ Res Public Health. 2024 Aug 18;21(8):1090. doi: 10.3390/ijerph21081090 (PMC11354165; doi:10.3390/ijerph21081090)
Supplement: Supplementary file 1 [file ijerph-21-01090-s001.zip › ijerph-3116721-supplementary.pdf]

International Journal of
Environmental Research and Public Health

MDPI

Table S1. ICD-10-CM code list.

Category	Codes
Obesity	E66; E66.0; E66.2; E66.3
Chronic Respiratory Disease	J40; J41; J41.0; J42; J43; J43.9; J44; J45; J45.0; J45.1; J45.9; J45.90; J45.909; J47; J47.9; J60; J61; J62; J62.8; J63; J64; J65; J66; J67; J67.9; J68.4; J70.1; J70.3; J98.4; K86.9
Serious Heart Conditions	I20; I21; I22; I23; I24; I25; I70; K76.1; I09.81; I11.0; I13.0; I26.09; I31.4; I50; I50.1; I50.2; I50.20; I50.22; I50.21; I50.23; I50.3; I50.30; I50.31; I50.42; I50.33; I50.4; I50.41; I50.32; I50.43; I50.81; I50.810; I50.812; I50.82; I50.9; I51.7; J81; J81.0; J81.1; P29.0; R09.2; A18.84; I01.0; I09.2; I30; I30.1; I30.8; I30.9; I31; I31.9; I32; M05.3; A36.8; A36.81; I01.2; I09.0; I40; I40.1; I40.8; I40.9; I41; I51.4
COVID-19 Infection	B34.2; B97.2; U07.1; U07.2; U071.1; U071.2; U071.4; U071.5; U08; U10; Z038PA1
HIV	B20; B20.0; B20.1; B20.2; B20.3; B20.4; B20.5; B20.7; B21.0; B21.1; B22.0; B22.2; B23.0; B24; B20.6; B20.8; B20.9; B21; B21.2; B21.3; B21.7; B21.8; B21.9; B22; B22.1; B22.7; B23.1; B23.2; B23.8
Lipid Abnormalities /Dyslipidemia	E75.240; E75.241; E75.242; E75.243; E75.248; E75.249; E75.3; E75.5; E75.6; E77; E77.0; E77.1; E77.8; E77.9; E78.1; E78.2; E78.3; E78.4; E78.49; E78.5; E78.6; E78.70; E78.79; E78.8; E78.81; E78.89; E78.9; E88.1; E88.2; E88.8; E88.89; M14.3
Chronic Kidney Disease	N03; N18; N18.9; N03.9; N26; N08; I12.0; I13.2; I13.1; I13; I13.9; N28.9; N19; N04; N05; N05.9; N04.9; N27; N27.9; E10.2; E11.2; E14.2
Immunodeficiencies	B20; B22; B22.0; B22.1; B22.2; B22.7; B23; B23.0; B23.1; B23.2; B23.8; B24; B26; B26.9; C38.4; D70; D70.9; D71; D76.1; D80; D80.0; D80.1; D80.3; D80.4; D80.5; D80.6; D80.7; D80.8; D80.9; D81; D81.0; D81.1; D81.2; D81.3; D81.30; D81.4; D81.5; D81.6; D81.7; D81.8; D81.89; D81.9; D82; D82.0; D82.1; D82.2; D82.3; D82.8; D82.9; D83; D83.0; D83.1; D83.2; D83.8; D83.9; D84; D84.0; D84.1; D84.8; D84.89; D84.9; D86; D89; D89.0; D89.1; D89.2; D89.3; D89.8; D89.89; D89.9; F02.4; G11.3; Q45.9; Q89.9
Hypertension	I10; I11; I11.0; I11.9; I12; I12.0; I12.9; I13; I13.0; I13.1; I13.2; I13.9; I15; I15.0; I15.1; I15.2; I15.8; I15.9; H35.03; O13
Type 1 and Type 2 Diabetes	E08; E09; E10; E10.0; E10.1; E10.2; E10.3; E10.36; E10.4; E10.5; E10.6; E10.69; E10.7; E10.8; E10.630; E11; E11.0; E11.1; E11.2; E11.3; E11.4; E11.5; E11.6; E11.69; E11.7; E11.8; E12.0; E12.1; E12.2; E12.3; E12.4; E12.5; E12.6; E12.7; E12.8; E13; E13.0; E13.1; E13.2; E13.3; E13.4; E13.5; E13.6; E13.69; E13.7; E13.8; E14; E14.0; E14.1; E14.2; E14.3; E14.4; E14.5; E14.6; E14.7; E14.8; E14.9; R73.9; G59.0; G63.2; H28.0; H34.2; H36.0; I70.2; M14.2
Polycystic Ovary Syndrome	E28.2
Secondary Amenorrhea	N91; N91.1; N91.2; N91.4; N91.5; N94.6
Heavy Menstrual Bleeding	N92.0; N92.1; N92.6; R58

Table S2. ATC code list.

Subgroup category	Codes
Hormonal Contraceptives for Systemic Use	G03AA; G03AB; G03AC
Androgens	G03BA; G03BB
Estrogens	G03CA; G03CB; G03CC; G03CX
Progestogens	G03DA; G03DB; G03DC
Androgens and Female Sex Hormones in Combination	G03EA; G03EB; G03EK
Progestogens and Estrogens in Combination	G03FA; G03FB
Gonadotrophins and Other Ovulation Stimulants	G03GA; G03GB

Antiandrogens	G03HA
Other Sex Hormones and Modulators of the Genital System	G03XA; G03XB; G03XC

Table S3. Association between COVID-19 vaccines doses and HMB and AM between first and second dose. (Crude Model).

	Heavy Menstrual Bleeding		Amenorrhea	
	**OR**	**95% CI**	**OR**	**95% CI**
Pfizer/BioNTech® *(Ref.)*				
AstraZeneca®	1.299	(1.141–1.471)	0.819	(0.726–0.919)
Janssen®	5.682	(5.253–6.142)	3.675	(3.427–3.937)
Moderna®	1.680	(1.552–1.817)	1.669	(1.572–1.771)

Total number of observations: 1172621
Data are number.
OR: Odds Ratio; 95% CI: 95% Confidence Interval

Table S4. Association between COVID-19 vaccines doses and HMB and AM between second and third dose. (Crude model).

	Heavy Menstrual Bleeding		Amenorrhea	
	**OR**	**95% CI**	**OR**	**95% CI**
Pfizer/BioNTech® *(Ref.)*				
AstraZeneca®	0.663	(0.610–0.718)	0.469	(0.437–0.505)
Moderna®	1.017	(0.977–1.058)	0.941	(0.912 - 0.971)

Total number of observations: 1063233
Data are number.
OR: Odds Ratio; 95% CI: 95% Confidence Interval
